# Disease-specific N-glycopeptides in serum of patients with oral squamous cell carcinoma

**DOI:** 10.1038/s41598-025-17339-w

**Published:** 2025-09-02

**Authors:** Tiialotta Tohmola, Sakari Joenväärä, Amy Dickinson, Antti Mäkitie, Suvi Silén

**Affiliations:** 1 Transplantation Laboratory, Haartman Institute and Helsinki University Hospital, University of Helsinki, PL 21 (Haartmaninkatu 3), Helsinki, 00014 Finland; 2 HUSLAB, Helsinki University Hospital, Helsinki, Finland; 3 Department of Otorhinolaryngology—Head and Neck Surgery, University of Helsinki and Helsinki University Hospital, Helsinki, Finland; 4 Research Program in Systems Oncology, Faculty of Medicine, University of Helsinki, Helsinki, Finland

**Keywords:** OSCC, Oral cancer, Tongue cancer, Mass spectrometry, Proteomics, N-glycoproteomics, Head and neck cancer, Oral cancer, Mass spectrometry, Proteomics

## Abstract

The incidence of oropharyngeal cancers is increasing due to human papilloma virus (HPV); however, this phenomenon does not explain the rising incidence of oral cancers, for which the reason remains unknown. These cancers are typically diagnosed at an advanced stage, which adversely affects the prognosis. Improved methods for early detection, such as blood-based biomarkers, could significantly improve patient outcomes. We investigated quantitative and qualitative differences in serum protein N-glycosylation between patients with oral squamous cell carcinoma (OSCC) and healthy controls using label-free mass spectrometry-based proteomics and N-glycopeptidomics. Twenty-five N-glycopeptides from seven different proteins were identified, all glycoforms associated with abundant serum proteins. While protein expression differences between OSCC and controls were modest (≤ 2-fold), certain glycoforms showed pronounced changes, with fold differences ranging from 7 to 33, highlighting their role as potential biomarkers. Importantly, the glycosylation patterns observed in OSCC were distinct from those previously reported in oropharyngeal cancer, suggesting a level of disease specificity. These observations support the future potential of N-glycosylation changes to serve as cancer-specific biomarkers.

**Supplementary Information:**

The online version contains supplementary material available at 10.1038/s41598-025-17339-w.

## Introduction

Cancers of the oral cavity are almost exclusively oral squamous cell carcinomas (OSCCs)^1^. Of all head and neck squamous cell carcinomas, OSCC – together with oropharyngeal squamous cell carcinoma (OPSCC) – is the most common cancer type^2^. In 2022, oral cancer (together with cancer of the lip) was the 16th most common cancer worldwide, with over 389,485 new cases and more than 188,230 deaths^3^. Moreover, oral cancer is nearly three times more common in males than females worldwide, and has a mortality rate of 48%^3^. Alcohol and smoking tobacco are considered risk factors for OSCC^2^.

In the Nordic countries, the incidence of tongue squamous cell carcinoma has multiplied 2.8-fold since 1960^4^. During the same period, the increase among young adults (ages 20 to 39) was over four-fold. This rising trend is expected to continue across all age groups, although the reasons for the increase remain unclear.

The diagnosis of OSCC is based solely on a histological biopsy of a visible tumor. OSCC typically starts to cause symptoms only when it has progressed to an advanced stage, delaying its detection and diagnosis, which significantly affects prognosis and survival rates. The overall 5-year survival rate is approximately 50%^2^. Late detection also leads to a higher likelihood of lymph node metastasis, and less commonly distant metastasis, further shortening survival times and increasing the chance of recurrence. About 65% of OSCC cases spread to lymph nodes, and 5–25% spread to distant organs^5,6^. The recurrence rate remains high, even for cases detected and treated early. Approximately 10–25% of early-stage OSCCs will recur; this increases to 40–60% in more advanced stages^7^. The prognosis of patients with recurrent tumors is poor, but having an early-stage primary tumor and a longer interval between carcinomas can improve the outcome^8^.

Glycosylation, a prevalent post-translational modification (PTM) of proteins, is influenced by various stress conditions, including cancer. This process is tightly controlled and non-template-driven, relying on the activity of glycosylating enzymes. Glycans impact numerous aspects of the function and physical properties of proteins, such as stability, folding, and solubility^9^. Glycosylation also plays a crucial role in protein secretion and transport, immune response and epitopes, and receptor interactions, among other functions^10^.

Since glycans are involved in every known hallmark of cancer, alterations in glycosylation during neoplastic processes make them potential biomarkers for diseases^11,12^. For example, prostate-specific antigen (PSA), a gold standard clinical marker for prostate cancer, demonstrates greater specificity for the disease when its glycosylation profiles are examined^13^. A low core fucosylation of PSA and high a2,3-sialic acid percentage in serum can distinguish high-risk prostate cancer from low-risk prostate cancer or benign prostatic hyperplasia^14^. Many other protein markers, such as alpha-fetoprotein (AFP), a biomarker of hepatocellular carcinoma (HCC), rely on the changes in their protein glycosylation in the detection of cancer. Serum levels of core-fucosylated AFP are significantly increased in individuals with HCC^15^. For these glycoproteins, their diagnostic specificity is due in part to abnormal glycosylation patterns, not just to changes in the overall protein levels, which highlights the importance of glycan research in the identification of new biomarkers.

In this study, we compared the sera of patients with OSCC of the tongue to the sera of healthy individuals using label-free serum proteomics and N-glycopeptidomics, to discover the possible differences in expression of glycoproteins between groups.

## Results

**Fig. 1:**
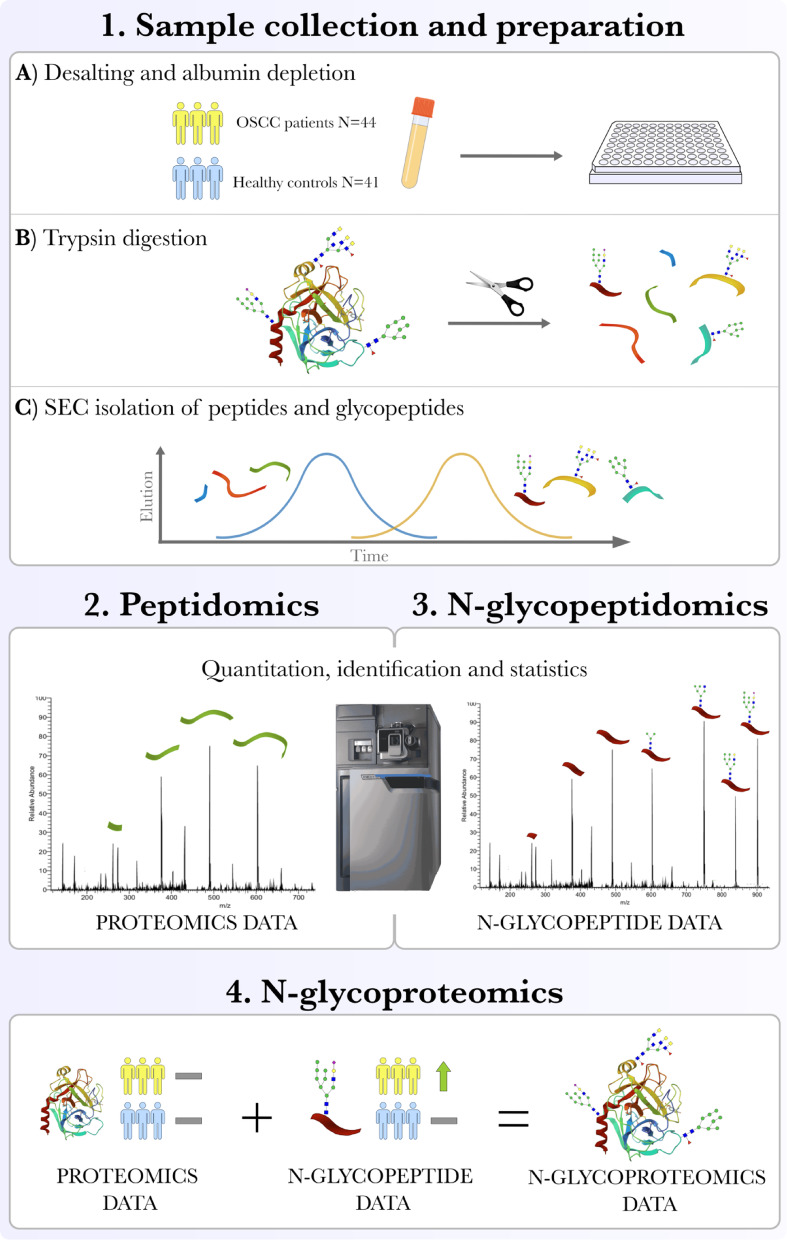
Study workflow. This figure outlines the overall study workflow. (**A**) Serum samples were collected from patients with OSCC (yellow) and healthy controls (blue), followed by desalting and albumin depletion of the samples. (**B**) Next, proteins were digested enzymatically using sequence-grade trypsin. (**C**) Peptides and N-glycopeptides were then separated and enriched using size exclusion chromatography (SEC) based on the size and shape of the peptides. Mass spectrometry was used for the in-parallel quantification and identification of peptides (2) and N-glycopeptides (3) to compare protein (proteomic data) and N-glycopeptide (N-glycopeptide data) expression levels between study groups. Following quantification and identification, statistical analysis was conducted to evaluate serum expression changes between the patients with OSCC and healthy controls. The graph presents an example of a peptide (2) and N-glycopeptide (3) fragmentation spectra. Finally, the N-glycoproteomics dataset (4) was created by combining proteomics and N-glycopeptide data. The graph provides an example of when a protein shows no difference in expression levels between OSCC patients and healthy controls, while its corresponding N-glycopeptide is overexpressed in OSCC patients (4). This integrated N-glycoproteomics approach enables determination of whether changes in glycan expression occur independently of protein expression.

We performed proteomics and N-glycopeptidomics in parallel using the same samples and sample processing to determine if the changes in glycosylation are linked to the protein expression or if they are independent of it, by comparing patients with OSCC to healthy controls. Firstly, we performed a standard proteomics experiment to quantify and identify the proteins (Fig. 1). Secondly, we quantified the glycopeptides and performed statistical analysis to identify glycopeptides that differed significantly between the groups. These statistically significant glycopeptides were targeted for fragmentation and identification in a separate LC-MS2 run.

We identified 25 glycan variants from seven different plasma proteins that were differentially expressed between the serum of patients with OSCC and the healthy controls. These glycoforms and their abundances are shown in (Table 1).

### Proteomics

The acquired raw MS data was collected and uploaded into Progenesis’ Proteinlynx Global Server (PLGS) with a maximum FDR of 1%. The data were mass corrected with internal E. coli standard protein Hi3, and the runs were aligned to correct for any drift in retention time. In total, 454 proteins (excluding albumin and standard protein Hi3) were identified and quantified without any additional filters applied to the dataset. The runs were normalized with Hi3 internal standard to correct for technical or experimental variation between the runs. The non-filtered ion data was plotted in a principal component analysis (PCA) on the peptide level in S1 Fig in the supplemental data.

After this step, a requirement of at least two uniquely sequenced peptides per protein was applied to the dataset, and the number of proteins was filtered to 207. The Benjamini-Hochberg procedure was applied to the whole protein dataset with a maximum FDR of 5%. The difference in expression levels of 133 proteins reached statistical significance after the Benjamini-Hochberg procedure.

### N-Glycopeptidomics

After the proteomic analysis, all the N-glycopeptide ions were quantitated with MS (Fig. 1). Next, only the glycopeptides that were statistically different between OSCC patients and controls were further targeted and analyzed with MS2 to determine the glycan composition and peptide sequence.

This MS2 analysis revealed 25 N-glycopeptides from seven different proteins (Table 1). All of the identified glycoforms were associated with abundant serum proteins, such as immunoglobulin heavy constant alpha −1 (IGHA1), gamma −1 and −2 (IGHG1 and IGHG2), haptoglobin (HPT) and alpha-1-acid glycoprotein −1 (A1AG1). Seven glycopeptides were excluded from the final set of N-glycopeptides, since we were not able to quantify the expression of their protein core.

When groups were compared, most of the glycoforms showed differences that were less than 2-fold. The biggest fold change was 33.27 of glycoform #4 attached to IGHA1 at site N144; IGHG1 site N180 with glycans #8 and #17 had fold changes of 8.09 and −7.67 (the negative value indicates that the expression levels were lower in serum of patients with OSCC). In all identified glycopeptides, the differences between the groups were significant (p-value < 0.05). Of the 25 glycopeptides subjected to MS2 analysis, the differences remained significant in 16 after applying the Benjamini-Hochberg procedure.

The N-glycan biosynthesis progresses from high mannose to hybrid to complex structures, which are more often sialylated and fucosylated. The structures of the glycans identified in this study were mainly complex or hybrid. Fifteen of the 25 identified glycans were sialylated and 15 were core-fucosylated. The expression of more complex sialylated or fucosylated glycans was slightly increased in the OSCC cohort.

### Principal component analysis

**Fig. 2:**
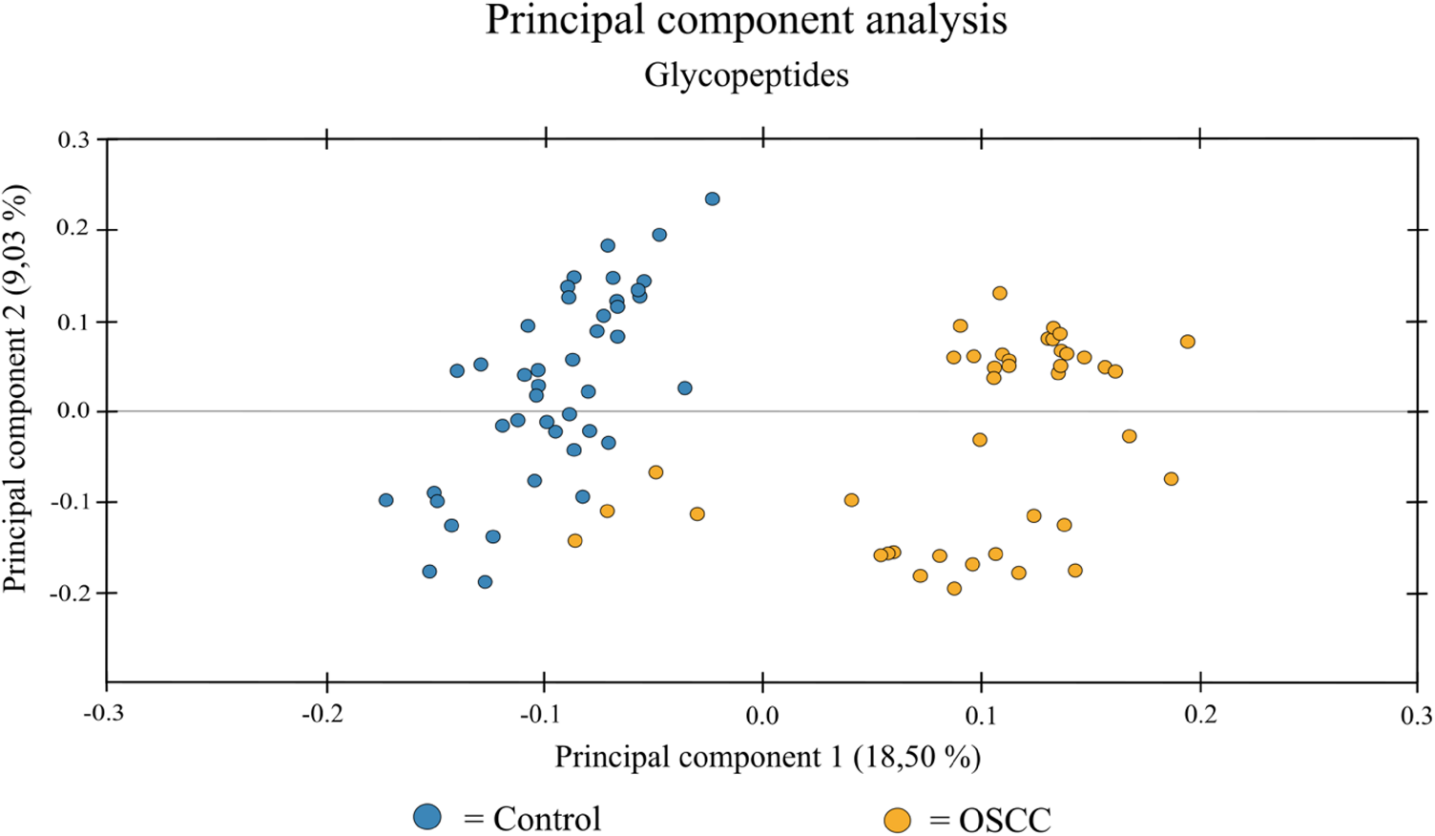
Principal component analysis of glycopeptides. All the N-glycopeptide ion data re-duced to two principal components (PC). Two different clusters are observed, with a minor overlap.

In the principal component analysis (PCA) analysis, we included all of the N-glycopeptide ions from the first MS quantitation step. The glycopeptide ion data were reduced to two principal components and plotted in (Fig. 2). Two different clusters can be seen, with a slight overlap. Interestingly, of the four overlapping individuals, all had tumors without lymph node metastases (N0).

### N-glycoproteomics

We combined N-glycopeptidomics and proteomics into N-glycoproteomics data, consisting of both glycan and amino acid chain abundances and glycan compositions, to discover whether the changes in glycan levels were dependent on protein levels. These comparisons are listed in (Table 1). Twenty-five N-glycopeptides had a corresponding protein found in the proteomic analysis. The protein expression fold changes between OSCC and controls were small regardless of their statistical significance. Meanwhile, some of the fold changes of the glycopeptide levels between groups were higher, leading to a conclusion that these changes in glycosylation were protein independent.

**Table 1:**
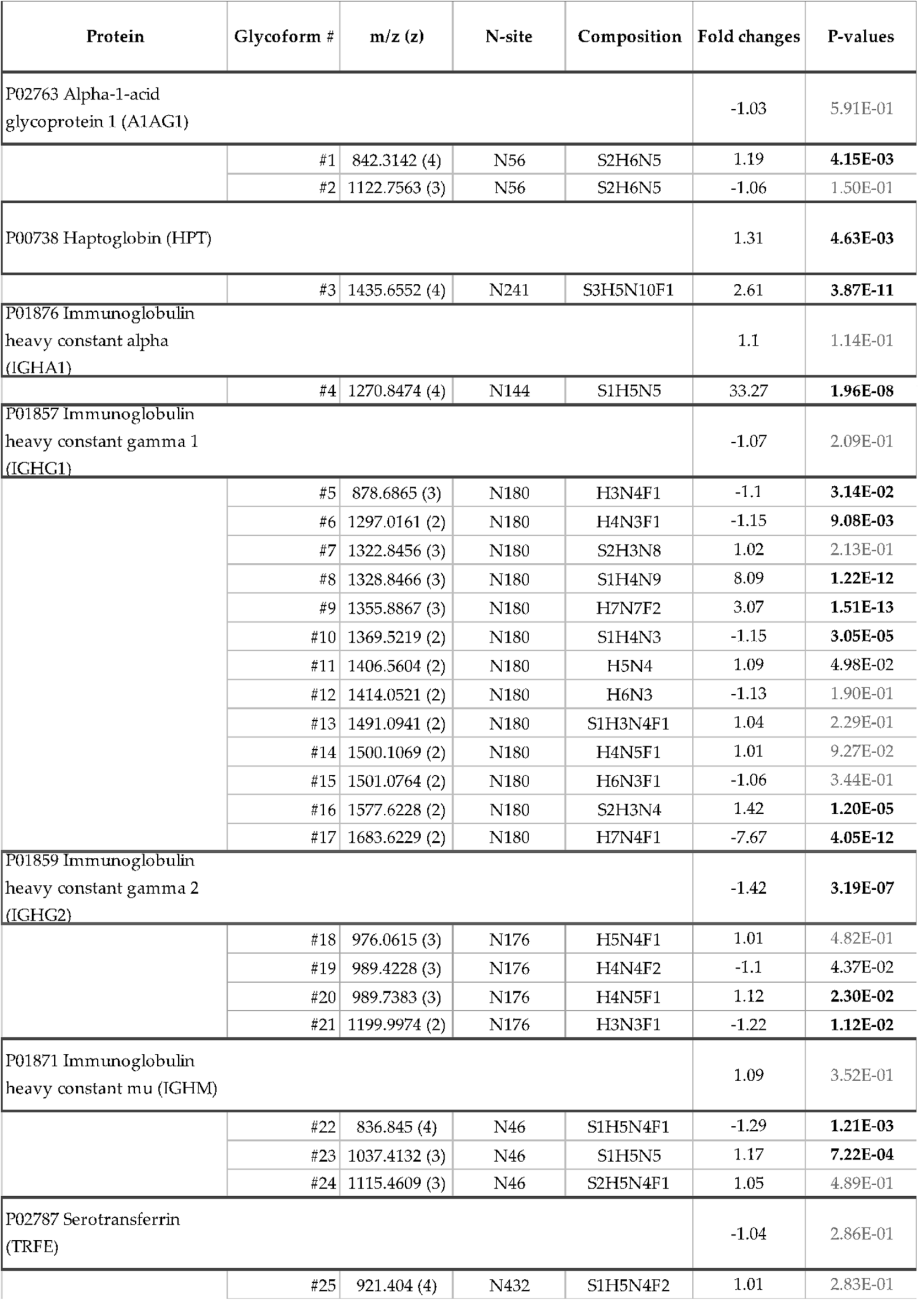
Identified and quantified N-glycoproteomics data. The N-glycoproteomics dataset was generated by integrating proteomics and N-glycopeptide data. Protein headers represent the protein backbone from which glycoforms were identified, and include the protein’s abbreviation, UniProt identifier, and full name. Each glycoform is assigned an identifier number (#1 to #25) and is characterized by its mass-to-charge ratio (m/z) and charge state (z). The N-site indicates the specific location on the protein backbone where the glycoform is attached. Glycan composition is also detailed, with the following abbreviations: S – sialic acid, H – hexose, N – N-acetyl-hexosamine, and F – fucose. The fold change represents the expression difference between OSCC patients and healthy controls. Student’s t-test p-values before multiple-testing correction are provided for each protein and glycoform. Statistically significant values after Benjamini-Hochberg correction (FDR < 0.05) are highlighted in bold. Values in black (but not bold) were significant before correction, while grey values were not statistically significant. For example, alpha-1-acid glycoprotein (A1AG1), identified by UniProt code P02763, contains two glycoforms (#1 and #2) at N-site N56.

In all seven proteins, the identified N-glycans were attached to only one N-glycan site per protein. In four proteins, multiple glycan variants with different compositions were found at the same site. The number of different glycan variants was highest in IGHG1. In IGHG2, all variants were structurally complex and had one or two fucoses attached to the glycan. A1AG1 had two double sialylated complex variants, and IGHM had three different variants with single or double sialylated complex glycans. Two of the IGHM complex variants were also fucosylated.

Two glycoproteins and their discovered glycovariants are discussed in greater detail below. These are IGHG1, with the most glycan variants found in this study and IGHA1, with the biggest glycan fold change. Both glycosylation sites have been previously reported in the literature^16–18^.

### IGHG1

For IGHG1, 13 different glycoforms were discovered with different glycan expressions at site N180 (Table 1). When comparing OSCC patients and healthy controls, expression level changes of the 10 glycopeptides did not differ from the protein expression. At the protein level, the fold change of IGHG1 between groups was −1.07, and was not statistically significant. Three glycopeptides (glycopeptide #8, #9 and #17) had a different expression compared to the corresponding protein. Glycopeptide #17 was under-expressed in the OSCC cohort and had a -7.67-fold change, whereas glycopeptides #8 and #9 were overexpressed compared to the control group, with a fold change of 8.09 and 3.07, respectively.

The IGHG1 glycopeptides included both hybrid and complex glycan compositions. Seven glycopeptides were sialylated and seven were fucosylated. There is no clear trend in differences between groups for glycan types within glycosylation site N180, either in their composition or complexity.

### IGHA1

For IGHA1, one glycoform was identified at N-site N144 (Table 1). The fold change of the protein expression of IGHA1 between groups was 1.1, which was not statistically significant. Glycopeptide #4 identified from the IGHA1 protein had a fold change of 33.27 between the OSCC and healthy controls, which was the biggest fold change of all the glycopeptides identified in this study. It has a complex glycan structure with monosialylation, and the composition corresponds with a biantennary and bisecting structure.

Post-hoc analysis of overlapping OSCC samples.

As there were four OSCC samples overlapping with the control samples in the glycopeptide PCA (Fig. 2), a post-hoc analysis of these samples was performed. A one-way ANOVA was used to test the statistical difference of the following three study groups: OSCC patients, healthy controls, and the four overlapping patients. Each group comparison was tested with Mann-Whitney for statistical differences.

Statistical analysis revealed that the four overlapping OSCC samples formed a significantly distinct group from the other OSCC samples and healthy controls. These four individuals represented patients with OSCC without lymph node metastases, and their serum glycopeptide levels seem to correspond more to those of the healthy individuals. The abundances of the top three glycopeptides with the highest fold changes in this study (#4, #8 and #17) had similar expression levels as in the controls. These fold changes are calculated and presented in S2 Table in the supplemental data.

### Comparison to other studies

We compared the results of this glycopeptide study to our previous study of oropharyngeal squamous cell carcinoma (OPSCC)^19^. In the earlier OPSCC study, the same MS technique was used to compare serum protein and glycopeptide changes between the sera of patients with OPSCC and healthy controls. OPSCC were also divided into subgroups based on the HPV status and the stage of the tumor, and comparisons were done separately for stage I-II and stage III-IV tumors.

Two glycopeptides present in the current study on OSCC were also discovered in the OPSCC study. Firstly, glycopeptide #4 on IGHA1 had the highest fold change of 33.27 between patients with OSCC and healthy controls. However, fold changes in OPSCC sera – regardless of the tumor’s HPV status and stage – were under three. Secondly, glycopeptide #18 in IGHG2 had no change in glycopeptide expression levels between groups in the present study. Meanwhile, in the OPSCC study, the same glycopeptide was 28.6-fold overexpressed in the stage III-IV HPV-positive cancer group.

The results of this study were also compared with our previously conducted OSCC study with multi-lectin chromatography without proteomics^20^. Glycopeptide #5 present in the current study was also discovered in the previous OSCC study in IGHG1. While the glycopeptide fold change in the current study did not change between the study groups, it was overexpressed 10.5-fold in the cancer group in the previous study.

## Discussion

Earlier detection of cancer would clearly improve the prognosis of the patients with OSCC. Protein glycosylation is known to be altered in malignant processes, and specific alterations could act as potential biomarkers for diagnosing cancer in the future. This study aimed to identify, quantitate and compare serum proteins and N-glycopeptides of the sera of patients with OSCC and healthy controls.

All identified glycovariants in this dataset were either hybrid or complex. These glycans are structurally more diverse in their sugar residue composition and are synthetized from the precursor high-mannose glycan later in the synthesis process. These two types of glycans consist of additional N-acetyl hexosamines, galactoses, fucoses and sialic acids. The expression of sialylated and/or fucosylated glycans were slightly increased in the OSCC group, a phenomenon that has also been observed in lung cancer and melanoma, and hypothesized to promote tumor progression^21^. It is possible that the further-processed hybrid and complex glycans found in sera of patients with OSCC are a part of the cell’s response to the disease.

In this study, we discovered that the change in the protein expression levels in IGHA1 does not explain the change in the glycan expression, which seems to be regulated separately. The N144-glycosylation site found in our study has been previously mentioned in studies on glycosylation in human urine, tear, and bile^16–18^. The glycan composition identified at site N144 in other studies was predominantly H5N4, which is a complex glycan^15,16^. The glycan composition found in this study has not been mentioned in the literature nor linked to any diseases previously. Thus, it warrants further study in other cancer cohorts to see whether it is present in other cancerous diseases or if it is OSCC-specific. In patients with OPSCC – another squamous cell carcinoma anatomically located in very close proximity to OSCC – this glycopeptide was not significantly overexpressed when compared to healthy controls^19^.

Of the seven proteins in this dataset, IGHG1 had the highest number of different glycovariants. In total, 13 glycovariants were found at the N180 glycosylation site. The expression levels of three glycovariants at this N-site had greater differences between groups, and were also independent of the protein expression. The N-linked glycan microheterogeneity at the N180 site was vast, and although only a few glycovariants had significant changes in their expression between the groups, this combination of glycans is putatively disease-specific and might serve as a disease biomarker in the future. Elevated expressions of IGHG1 N180-site glycopeptides have also been found in our previous OSCC study based on lectins^20^. In that study, eight identified glycan compositions were over 5-fold overexpressed in the cancer group, four of which were over 10-fold overexpressed. However, without proteomic analysis, it is not clear whether the glycopeptide levels were dependent on the protein level. Lectin enrichment targets specific monosaccharides and structures, while size exclusion relies on the shape and size of molecules. This accounts for the different results observed between these studies. Multiple N180-site glycopeptide compositions have also been represented in a glycoproteomic study of colorectal cancer^22^. The IGHG1 glycopeptides identified as #5, #6, #11, and #14 in our study were also detected in the serum of colorectal cancer patients and healthy controls. Notably, glycopeptide #5 was proposed as a potential early diagnostic marker for colorectal cancer by Liu S. et al. This glycopeptide was also discovered to be over 10-fold overexpressed in our previous OSCC study done with lectins^20^. In this current study, expression in glycopeptide #5 was similar between patients and healthy controls. The differences in fold changes between the studies could be explained by the different glycopeptide isolation methods used.

We compared the results of the current study to our previous work on OPSCC done with the same workflow method^19^, which revealed two identical glycopeptides identified in both studies. However, the fold changes did not follow the same trend. The difference between OSCC and OPSCC could be explained by the different anatomical location and/or the biological aetiology of the two cancers. OSCC derives from the mucosal squamous stratified epithelium in the oral cavity, while OPSCC arises in the oropharynx, including the flattened non-keratinized or parakeratinized squamous epithelium of the tonsils and base of the tongue. In addition, almost half of OPSCCs in Europe are caused by HPV infection, a phenomenon not seen in OSCC, possibly further explaining the different results between these cancers. Patients with HPV-positive OPSCC expressed one IGHG2 glycoform (#18) almost 30-fold compared with patients with OSCC – a tumor that often lacks HPV-related changes even when HPV DNA is present in the tumor^19,23^.

The limitations of this study include methodological constraints. While we have analyzed highly abundant glycoproteins, detecting the low-abundant proteins and their glycans require more advanced protocols and instruments due to their low-intensity nature.

## Conclusions

N-glycopeptide expression levels can be altered in a specific manner in different diseases, including cancers. Changes in serum proteins can be disease-specific, but the fold changes tend to remain small. For their part, serum glycopeptide-level alterations are of a larger magnitude and easier to detect as potential biomarkers of the studied physiological condition.

This study discovered statistically significant changes in the expression of both proteins and N-glycopeptides. While the difference in protein expression levels between the sera of patients with OSCC and healthy controls was at most two-fold, a few glycovariants had fold differences from 7 to 33 between the groups. Most of the identified glycopeptides were different from those identified in our previous N-glycoproteomic study of OPSCC. However, two identical glycopeptides were found in both studies, although both of their expression levels differed around 30-fold between the cancers.

We believe these data support the possibility of using glycoproteins as disease-specific diagnostic biomarkers in the future. However, to gain access to more comprehensive and comparable data, it is necessary to continue developing the technological instrumentation as well as standard methods in the field of glycoproteomics.

## Materials and methods

### Patient and Sera samples

The sample cohort consisted of 41 serum samples of OSCC patients and 44 age- and gender-matched control serum samples. The OSCC serum samples were collected from pre-treatment OSCC patients during 1.1.2015–31.12.2019 at the Department of Otorhinolaryngology, Head and Neck Surgery, Helsinki University Hospital, Helsinki, Finland. Written informed consent was obtained from all patients. The study was conducted in accordance with the Declaration of Helsinki, and approved by the Institutional Research Ethics Committee of Helsinki University Hospital (Dnr: 64/13/03/02/2014).

The patient group included 24 males and 17 females, aged 24 to 97 years old. Any patients with coexisting inflammatory diseases, history of previous cancer within 18 years, concurrent cancers and/or subsequent second primary cancer were excluded. The 44 control samples were collected and provided by the Finnish blood service in 2020. Among the OSCC patients, 29% of tumours were classified as stage I, 22% as stage II, 24% as stage III and 24% as stage IV^24^ In 61% of the patients, the cancer had not spread to lymph nodes, representing N0 tumours. Twenty-four patients were reported to have a history of smoking. More detailed information about the patient demographics can be found in Table S1 in the supplemental data.

Sample preparation.

### Sample collection

The 41 blood samples collected from patients diagnosed with OSCC (tongue) and 44 control samples were left to clot at room temperature. To separate the serum, the clotted samples were centrifuged at 1,000 g in 4 °C for 10 min (Model 5810R; Eppendorf AG, Germany). An aliquot of the serum was stored at -70 °C until further use.

### Desalting

Using Zeba Spin desalting plates (96well 7k #89808; Thermo Fisher Scientific Ltd, MA, USA), the serum samples were desalted from excess salts and other small molecular contaminants. To ensure maximal albumin binding in the following step of the protocol, the buffer in the plate was exchanged to 5 mM Tris with 75 mM NaCl (pH ~ 7–8) (Trizma^®^ base, #T1503; Sigma-Aldrich, MO, USA) (Sodium chloride, #31434; Honeywell International Inc. NC, USA) according to the protocol. 90 µl of serum was added to the plate and centrifuged at 1,000 g for two minutes at room temperature (Model 5810R; Eppendorf AG, Germany). The desalted serum flow-through was collected into new 1.5 ml Eppendorf tubes (Protein LoBind^®^ Tubes #0030108116, Eppendorf AG, Germany) and stored at -20 °C until further use.

### Albumin depletion

Desalted serum was processed with a PierceTM Albumin Depletion Kit (#85160; Thermo Fisher Scientific Ltd, MA, USA) to remove albumin. 400 µl of the resin slurry was used per sample, and washed once according to the protocol. 50 µl of desalted serum was applied on top of the resin, and incubated for two minutes. The sample was centrifuged at 12,000 g for one minute at room temperature (Model 5415R; Eppendorf AG, Germany), and the flow-through was re-applied to the resin. It was then incubated and centrifuged again. Next, the resin was washed three times with 50 µl of wash buffer, and the washes were combined with the albumin-depleted serum.

### Bradford assay and sample aliquot

The protein concentration of the albumin-depleted samples was measured with a Bradford reagent (PierceTM Coomassie Plus (Bradford) Assay Kit #23236; Thermo Fisher Scientific Ltd, MA, USA). Bovine serum albumin (BSA) in a series of dilutions was used as a standard, with the wavelength set to 595 nm (Sense microplate reader, model type 425 − 301; Hidex Oy, FIN). An aliquot of 380 µg of total protein was dried from each sample with a SpeedVac vacuum concentrator (Model DNA120; Savant Systems LLC, MA, USA). Dried samples were stored at -20 °C until further use.

### Trypsin digestion

Sample proteins were digested with the aid of mass spectrometry suitable agent RapiGest SF Surfactant (#186002123; Waters Ltd Milford, MA, USA). The proteins were first denatured by resuspending the dried aliquot 0.2% RapiGest SF solution in 50 mM ammonium bicarbonate (#09832; Sigma-Aldrich, MO, USA). The samples were vortexed to resuspend the pellet and then further denatured in a thermomixer (Thermomixer comfort; Eppendorf Ltd, DE) set to 99 °C for 10 min with a small hole in the tube lid.

Denatured sample proteins were reduced with dithiothreitol (DTT, #V315B; Promega corp. WI, USA) at a final concentration of 5 mM and incubated in a 60 °C water bath for 30 min. After reduction, alkylation of sample proteins was performed by adding iodoacetamide (IAA, #57670; Sigma-Aldrich, MO, USA) at a final concentration of 15 mM following incubation at room temperature in darkness for 30 min. Denatured, reduced and alkylated proteins were then digested with sequence grade recombinant trypsin (Trypsin Gold, Mass Spectrometry Grade, #V5280; Promega corp. WI, USA) resuspended in 33 mM acetic acid. One µg of trypsin was added for every 100 µg of sample and incubated at 37 °C in darkness for 18 h.

After the overnight digestion, the samples were subjected to an acid treatment to remove RapiGest SF surfactant. Trifluoroacetic acid (TFA) (#302031; Sigma-Aldrich, MO, USA) was added to the samples at a final concentration of 0.5% and incubated for 45 min at 37 °C. As a result of adding the acid, the pH of the solution decreased below 2, which caused the surfactant molecule to degrade into two product molecules. The insoluble product molecules were removed by centrifuging at 16,000 g at 8 °C for 5 min (Model 5415R; Eppendorf Ltd, DE). The supernatant including the digested proteins was collected and dried with a SpeedVac vacuum concentrator (Model DNA120; Savant Systems LLC, MA, USA).

### SEC

Size exclusion chromatography was used to separate digested proteins into two fractions: a peptide and a glycopeptide fraction. The chromatographic protocol was prescreened to find optimal elution patterns for both fractions. The peptide fraction was later used for proteomic analysis, and the N-glycopeptide fraction for N-glycopeptidomic analysis.

The dried digested samples were resuspended with 55 µl of 0.1% formic acid (FA, #5.33002; Sigma-Aldrich, MO, USA) and vortexed until the pellet was resuspended. The samples were sonicated in a water bath for 5 min to dissolve the possible aggregates. After this, the samples were centrifuged at 10,000 g at 18 °C for 10 min to pellet down any remaining aggregates. A 50 µl aliquot of the sample was injected to a size exclusion column (SuperdexTM 30 Increase 3.2/300 #29-2197-58; GE Healthcare, IL, USA). The isocratic flow rate for the column was 0.08 ml/min with a mobile phase of 0.1% FA. Glycopeptide and peptide fractions were collected, and the column was washed for 30 min between samples. 100 µl of the peptide fraction and 270 µl of the glycopeptide fraction was aliquoted and dried with a SpeedVac vacuum concentrator (Model SPD121P; Thermo Fisher Scientific Ltd, MA, USA). The collected leftover fractions were stored at −70 °C.

### Peptide assay

The dried peptide aliquots were resuspended, and the peptide concentration was measured with Pierce^™^ Quantitative Fluorometric Peptide Assay (#23290; Thermo Fisher Scientific Ltd, MA, USA). 50 µl of 0.1% FA (#5.33002; Sigma- Aldrich, MO, USA), 2% acetonitrile (ACN, #1.00029; Sigma-Aldrich, MO, USA) in water was added to the samples, vortexing briefly and sonicating in a water bath for 5 min. 10 µl of the sample was used for the peptide assay.

### Preparation of MS samples

After measuring the peptide concentration, the proteomic samples were adjusted to a final concentration of 125 ng/µl in the MS vial and spiked with internal Hi3 *E. coli* standard (#186006012; Waters Ltd, Milford, MA, USA) at a final concentration of 50 fmol per injection in the mass spectrometer. Glycopeptide fractions were resuspended in 50 µl of 0.1% FA (#5.33002; Sigma- Aldrich, MO, USA), 2% acetonitrile (ACN, #1.00029; Sigma-Aldrich, MO, USA) in water and vortexed and sonicated for 5 min. 25 µl of the glycopeptide mixture was used in the MS vial and the leftover was stored at -70 °C.

### Mass spectrometry

#### Proteomics and glycopeptidomics: calibration and UPLC gradient

The mass spectrometry system calibration was performed with sodium iodide at a concentration of 2 µg/µl in 50% 2-propanol over a mass range of 50–2500 m/z. The gradient was 0.1% formic acid (#5.33002; Sigma-Aldrich, MO, USA) in water as buffer A and 0.1% formic acid in acetonitrile (#1.00029; Sigma-Aldrich, MO, USA) as buffer B. The sample was loaded into the trapping column with a flow of 8 µl/min and 2% concentration of buffer B. The following linear analytical gradient curve is pictured in (Table 2). The analytical flow rate was 200 nl/min and leucine enkephalin (556.2771 Da, C25H37O7) in 50% acetonitrile and 0.1% formic acid was infused from a secondary probe with a flow rate of 300 nl/min for post-acquisition.

**Table 2:** Analytical gradient table. The gradient between the timepoints was linear.

Time (min)	B%
0	2
1	2
75	45
80	90
85	50
87	90
90	2
100	2

#### Proteomics raw data collection: UDMSE

The data was collected with data independent acquisition (DIA) mode. Samples were analysed with the nano-UPLC and ultra definition mass spectrometry (UDMSE) method in resolution mode and positive mode to identify and quantify peptides. 125 ng of peptide mixture was injected into Synapt G2-Si coupled to nano-ESI ion source and UPLC nanoACQUITY from Waters Ltd (Milford, MA, USA). The trapping column used was a 180 μm x 20 mm C18 nanoACQUITY trapping column (#186006527; Waters Ltd, Milford, MA, USA) with 5 μm particle size and 100 Å pore size, and the analytical column was a 75 μm x 250 mm C18 reverse phase nanoACQUITY column (#186003815; Waters Ltd, Milford, MA, USA) with 1.7 μm particle size and 300 Å pore size. The data collection range was 100–2000 m/z, and 10% of the samples were run in triplicate, resulting in %CV of 2.9. IMS wave velocity was 650 m/s with 1 s scan time. The peptide CID was done in the transfer cell where high collision energy was altered and optimised for the precursors based on their drift-time in IMS.

#### Proteomics quantitation and identification

The acquired raw data was imported to Progenesis QIP software (Nonlinear Dynamics Ltd, Milford, MA, USA, version 4.2.7207.22925) and was mass corrected with lock mass ion leucine enkephalin (556.2771 Da) (#186006013; Waters Ltd, Milford, MA, USA). ProteinLynx Global SERVERTM (PLGS; Waters Corp., Milford, MA, USA, v3.03) was used to identify peptides from the MS^E^-data, and the mass corrected data was first aligned and then normalised with Hi3 *E. coli* standard (#186006012; Waters Corp., Milford, MA, USA). For the identification parameters, 2 trypsin miscleavages were allowed and FDR was set maximally to 1%. UniProt human FASTA sequences (UniProt UP000005640, 9606 - Homo sapiens, genome assembly GCA_000001405.27 from Ensembl, gene count 20 596, last modified January 15th 2020, added sequence P632851⁄2CLPB_ECO057) was used to identify peptides. Ions with a charge greater than 5 and errors over +/- 10 ppm were excluded. Quantification of the data was done using the Hi-N label free method with relative quantitation using non-conflicting peptides. The final dataset was formed after excluding Hi-3 standard, albumin and proteins with less than two unique peptides. Cysteine alkylation and variable methionine oxidation were included as fixed modifications. Principal component analysis PCA was done for the non-filtered peptide ion dataset excluding albumin and Hi3 *E. coli* standard, and plotted using two principal components.

#### Proteomics statistical analysis

The dataset of proteins with two or more unique peptides was tested for normality with Shapiro-Wilk, skewness and kurtosis, and the majority of the protein expressions were assumed to follow normal distribution. Student’s *t*-test with a p-value of 0.05 was chosen, as the dataset had two independent groups. Student’s *t*-test passed proteins were continued to the Benjamini-Hochberg procedure, which was used as a multiple-testing correction. This was calculated by ranking the p-values with a false discovery rate of 5%.

#### Proteomics network analysis

Pathway analysis tool ingenuity pathway analysis (IPA; QIAGEN Inc. Germany) was used to identify upregulated and downregulated pathways and networks present in the data. 207 proteins that passed the Student’s *t*-test (*p* < 0.05) were imported to the analysis tool. All canonical pathways within the imported protein set and the most enriched networks between direct interactions of the data set proteins were concluded from the analysis.

#### N-glycopeptidomics Raw data collection: MS^E^

The analytical gradient was identical in both analyses. The analytical flow rate for MSE and glycopeptide identification and fragmentation were 0.200 µl/min, and 1 µl of the sample was injected. Both quantitation and identification of N-glycopeptides were acquired with sensitivity mode in positive polarity.

N-glycopeptide quantification was done with MSE with a mass range of 50–2500 Da and an acquisition time of 0–100 min. The collision energy for the precursors was ramped from 14 eV (low energy) to 44 eV (high energy) in the trap cell. 1 s scan time was used, and 10% of the samples were acquired in triplicate.

#### N-glycopeptidomics: statistics

The non-linear and branched structures of N-glycopeptides require isolated fragmentation to identify them accurately. The raw MSE data was uploaded for Progenesis QIP (Nonlinear Dynamics Ltd, Milford, MA, USA, version 4.2.7207.22925) for lock mass correction and to exclude identified peptides from future fragmentation. In order to choose and target relevant glycopeptides for fragmentation, the dataset needs to consist of mainly said ions. We attempted to enrich N-glycopeptides and refined the dataset by removing ions that did not meet the criteria. Ions with lower than 500 m/z and ions with a mass lower than the theoretical minimal size of a N-glycopeptide of 1300 Da were removed from the dataset. Ions with charges of + 2 to + 5 were included. The data was not assumed to be normally distributed and was tested with Mann-Whitney U-test with a p-value of 0.05. To avoid false positives, the Benjamini-Hochberg procedure was applied to the dataset with an FDR of 5%. The statistically different glycopeptides between the groups were selected for fragmentation. Fragmentation was performed from three samples with the highest intensity of each glycopeptide.

#### N-glycopeptidomics: targeted CID fragmentation

Fragmentation of the N-glycopeptides was done with MS2 FAST DDA in positive sensitivity mode. The mass range was 50–2500 Da for the fragmentation and scan time of 1 s. In FAST DDA, collision energy was ramped from 20 to 60 V in transfer. The MS2 spectra was uploaded to MassLynx 4.1 software (Waters Corp., Milford, MA, USA) and manually deconvoluted using the MaxEnt3 module. The peak lists were saved as .pkl files.

#### N-glycopeptidomics: identification with glycopeptide ID

The peak lists were uploaded and analysed with Glycopeptide ID software. The software is an open-access web service developed by Applied Numerics Ltd (Helsinki, Finland) in collaboration with the University of Helsinki for the analysis of intact N-glycopeptide CID LC-MS2 data. Firstly, the peak lists were searched against an in-house generated peptide database (UniProt proteome ID UP000005640, Genome assembly GCA_000001405.27, gene count 20,596 and protein count 74,823) containing tryptic peptides from human proteome containing N-glycosylation consensus sequence. In the peptide identification process, the same modifications and mass errors were used as in the proteomics. In the peptide search parameters, two tryptic miscleavages, variable methionine oxidation modification and fixed cysteine carbamidomethyl were allowed. Secondly, a matching de novo monosaccharide composition was determined based on the mass of the glycan, which was calculated by subtracting the theoretical mass of the peptide sequence from the total mass of the glycopeptide. Lastly, all the glycan compositions were manually curated to be possible by N-glycosylation enzymatic pathways.

#### Post-hoc analysis of overlapping OSCC samples

After the initial N-glycopeptidomic analysis, a post-hoc analysis of overlapping OSCC samples was done. We focused on the identified and quantified glycopeptides and divided their expressions into three groups: OSCC, controls, and overlapping N0 samples. The statistical difference of multiple study groups was tested with one-way ANOVA, where the three groups were independent and normal distribution was assumed based on Skewness and Kurtosis. Next, the fold changes were calculated, and statistical differences were tested with Mann-Whitney for each group comparison.

## Supplementary Information

Below is the link to the electronic supplementary material.

Supplementary Material 1

## Data Availability

The mass spectrometry proteomics data have been deposited to the ProteomeXchange Consortium via the PRIDE^25^ partner repository, with the dataset identifier PXD055139 and 10.6019/PXD055139.The mass spectrometry N-glycopeptidomics data have been deposited to the ProteomeXchange Consortium via the PRIDE^25^ partner repository, with the dataset identifier PXD055600.

## Abbreviations

A1AG1: Alpha-1-acid glycoprotein 1
AFP: Alpha-fetoprotein
DDA: Data-dependent acquisition
DIA: Data-independent acquisition
ESI: Electrospray ionization
HCC: Hepatocellular carcinoma
HPT: Haptoglobin
HPV: Human papilloma virus
IGHA1: Immunoglobulin heavy constant alpha −1
IGHG1/2: Immunoglobulin heavy constant gamma −1, −2
IGHM: Immunoglobulin heavy constant mu
LC-MS: Liquid chromatography mass spectrometry
MS: Mass spectrometry
OPSCC: Oropharyngeal squamous cell carcinoma
OSCC: Oral squamous cell carcinoma
PCA: Principal component analysis
PSA: Prostate-specific antigen
PTM: Post-translational modifications
SEC: Size exclusion chromatography
TRFE: Serotransferrin
UDMSE: Ultra-definition mass spectrometry
UPLC: Ultra-performance liquid chromatography
