# Supplementary material for: Disease-specific N-glycopeptides in serum of patients with oral squamous cell carcinoma
Source: Sci Rep. 2025 Sep 2;15:32276. doi: 10.1038/s41598-025-17339-w (PMC12402464; doi:10.1038/s41598-025-17339-w)
Supplement: Supplementary file 1 — Supplementary Material 1 [file 41598_2025_17339_MOESM1_ESM.pdf]

S1 Table. Patient demographics. Tumour stage was reported according to the size of the primary tumour, as well as its invasion to nearby structures (T), involvement of locoregional lymph nodes (N), and distant metastases (M) (18). Patients with coexisting inflammatory diseases and/or history of another cancer within 18 years were excluded.

Age	Gender	Stage	TNM classification
24	M	Stage II	T2N0M0
34	M	Stage I	T1N0M0
34	F	Stage III	T2N1M0
36	F	Stage I	T1N0M0
39	M	Stage III/IV	T1N1-2M0
42	F	Stage III	T2N1M0
43	M	Stage III	T1N1M0
44	F	Stage III	T3N0M0
44	F	Stage III	T2N1M0
45	M	Stage III	T3N0M0
46	M	Stage IV	T3N2M0
48	M	Stage I	T1N0M0
50	M	Stage IV	T4N2M0
50	F	Stage II	T2N0M0
54	M	Stage III	T3N1M0
54	F	Stage IV	T2N3M0
56	M	Stage I	T1N0M0
58	M	Stage I	T1N0M0
59	M	Stage I	T1N0M0
59	F	Stage I	T1N0M0
63	M	Stage IV	T4N1M0
64	M	Stage II	T2N0M0
64	M	Stage I	T1N0M0
66	F	Stage III	T3N0M0
66	M	Stage I	T1N0M0
67	F	Stage IV	T4N1M0
69	M	Stage II	T2N0M0
69	F	Stage II	T2N0M0
69	M	Stage IV	T4N2M0
69	M	Stage IV	T2N2M0
70	M	Stage IV	T3N3
73	F	Stage II	T2N0

73	F	Stage I	T1N0M0
74	M	Stage III	T2N1M0
74	M	Stage III	T2N0M0
74	M	Stage IV	T2N2M0
75	M	Stage II	T2N0M0
76	F	Stage II	T2N0M0
78	F	Stage I	T1N0M0
79	F	Stage I	T1N0M0
97	F	Stage II	T2N0M0

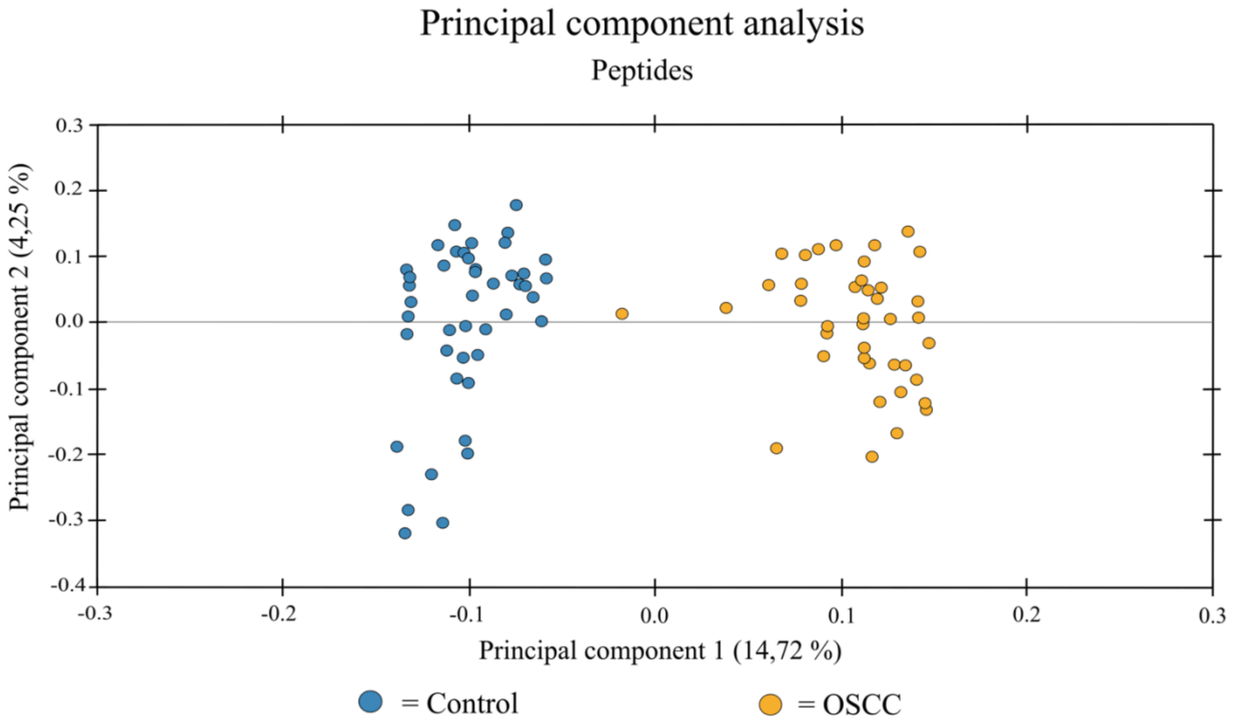

S1 Fig. Principal component analysis of peptides. On the peptide level, the data was reduced to two principal components (PC), and two separate clusters are seen. The data contains expressions of all ions from the raw data except for albumin and Hi3 *E. coli* standard.

S2 Table. Post-hoc analysis of the glycopeptide level changes in "control-like OSCCs" vs other OSCCs vs healthy controls. In this post-hoc analysis, the samples were

reorganised into three groups instead of two. In the original glycopeptide PCA (Fig 2), four OSCC samples were overlapping with the controls. The clinical information of these patients revealed that all had OSCC tumours without lymph node metastases, with a TNM classification of N0. These four samples were removed from the OSCC group and analysed as a separate group, referred to as “control-like OSCC”. To compare the glycopeptide level changes between the control-like OSCCs, other OSCCs and healthy controls, one-way analysis of variance (ANOVA) was performed, identifying 21 glycopeptides with different expression levels ($p < 0.05$) between the groups. Pairwise comparisons were then performed between all three groups to determine the fold changes of expression levels, the statistical significance of which was tested with a Mann-Whitney test ($p < 0.05$ in bold in the table). Two ions (1328.8466 m/z and 1270.8474 m/z) had significantly different expression levels in the control-like OSCC group compared with the rest of the OSCC samples and closely resembled those of the control group. This probably contributed to their clustering with the controls in the original glycopeptide PCA (Fig 2).

ANOVA One-Way	FOLD CHANGE			Mann-Whitney			Glycosylation site			
**P-value**	**OSCC vs Control**	**OSCC vs Control-like OSCC**	**Control vs Control-like OSCC**	**OSCC vs Control**	**OSCC vs Control-like OSCC**	**Control vs Control-like OSCC**	**Protein**	**N-site**	**Composition**	**m/z (z)**
**1.22E-41**	8.86	10.00	1.13	**8.50E-15**	**6.20E-04**	2.17E-01	Immunoglobulin heavy constant gamma 1 (IGHG1)	180	S1H4N9	1328.8466 (3)
**1.15E-17**	36.78	41.06	1.12	**4.74E-10**	**3.93E-03**	2.06E-01	Immunoglobulin heavy constant alpha (IGHA1)	144	S1H5N5	1270.8474 (4)
**5.10E-12**	3.16	1.64	-1.93	**7.57E-13**	6.48E-02	**2.48E-02**	Immunoglobulin heavy constant gamma 1 (IGHG1)	180	H7N7F2	1355.8867 (3)
**5.85E-12**	-7.11	-2.35	3.02	**1.80E-11**	5.94E-02	**2.96E-02**	Immunoglobulin heavy constant gamma 1 (IGHG1)	180	H7N4F1	1683.6229 (2)
**1.82E-08**	2.77	2.52	-1.10	**7.64E-12**	**2.00E-03**	2.06E-01	Haptoglobin (HPT)	241	S3H5N10F1	1435.6552 (4)
**3.88E-07**	-1.53	-1.98	-1.30	**1.65E-06**	**1.31E-03**	8.80E-02	Immunoglobulin heavy constant gamma 1 (IGHG1)	180	S2H3N4	1577.6228 (2)
**3.75E-05**	1.24	1.49	1.21	**2.56E-04**	**1.65E-02**	1.15E-01	Alpha-1-acid glycoprotein 1 (A1AG1)	56	S2H6N5	842.3142 (4)
**5.99E-04**	1.02	-1.35	-1.37	1.12E-01	**2.00E-03**	**3.60E-03**	Immunoglobulin alpha Fc receptor (FCAR)	65	H4N7	1355.5326 (2)
**6.69E-04**	2.04	2.33	1.14	**2.06E-05**	**2.05E-02**	4.02E-01	Nucleus accumbens-associated protein 1 (NACC1)	353	S3H7N4F1	1413.6303 (4)
**7.77E-04**	-1.15	-1.00	1.14	**9.77E-05**	4.91E-01	**2.48E-02**	Immunoglobulin heavy constant gamma 1 (IGHG1)	180	S1H4N3	1369.5219 (2)
**1.05E-03**	-1.19	-1.47	-1.24	**2.89E-03**	**2.00E-03**	6.60E-02	Immunoglobulin heavy constant gamma 1 (IGHG1)	180	H4N3F1	1297.0161 (2)
**1.59E-03**	1.24	1.02	-1.22	**9.05E-05**	4.39E-01	**3.51E-02**	Immunoglobulin alpha Fc receptor (FCAR)	65	S2H3N8	1149.1075 (3)
**6.18E-03**	1.82	1.40	-1.31	**1.60E-04**	2.91E-01	7.64E-02	Apical endosomal glycoprotein (AEGP)	203	S1H4N6F1	1465.6226 (2)
**8.77E-03**	-1.26	1.03	1.29	**1.90E-03**	4.74E-01	1.08E-01	Contactin-associated protein-like 3B (CNT3B)	1278	S1H8N7	1248.4836 (3)
**9.49E-03**	-1.43	-2.74	-1.92	**1.39E-03**	**5.76E-03**	**1.89E-02**	Immunoglobulin heavy constant gamma 2 (IGHG2)	176	H3N3F1	1199.9974 (2)
**1.35E-02**	-1.16	-1.49	-1.29	1.34E-01	**1.84E-02**	9.42E-02	Immunoglobulin heavy constant gamma 1 (IGHG1)	180	H6N3	1414.0521 (2)
**1.43E-02**	1.13	-1.28	-1.45	**1.42E-02**	**4.54E-02**	**7.74E-03**	Immunoglobulin gamma-1 heavy chain (IGG1)	299	H4N4F1	1412.5525 (2)
**1.85E-02**	1.19	1.20	1.01	**3.55E-03**	6.48E-02	4.77E-01	Sickle tail protein homolog (SKT)	1539	H7N5	1248.0332 (4)
**3.09E-02**	-1.12	-1.12	-1.00	**1.46E-02**	9.01E-02	1.65E-01	Immunoglobulin heavy constant gamma 2 (IGHG2)	176	H4N4F2	989.4228 (3)
**3.26E-02**	1.00	-1.42	-1.41	1.18E-01	**3.09E-02**	**3.22E-02**	Immunoglobulin heavy constant gamma 1 (IGHG1)	180	H4N5F1	1500.1069 (2)
**3.57E-02**	1.19	1.01	-1.18	**6.56E-04**	4.91E-01	7.64E-02	Immunoglobulin heavy constant mu (IGHM)	46	S1H5N5	1037.4132 (3)
